# False Positive Positron Emission Tomography/Computed Tomography (PET/CT) Requiring Biopsy for Proper Staging of Lung Cancer

**DOI:** 10.7759/cureus.34497

**Published:** 2023-02-01

**Authors:** Tanner Allen, John Hilu, Muhammad Amin

**Affiliations:** 1 General Surgery, Beaumont Health, Dearborn, USA; 2 Cardiothoracic Surgery, Beaumont Health, Dearborn, USA

**Keywords:** false positive pet scan, lung cancer staging, metastatic lung cancer, biopsy, lung cancer

## Abstract

Lung cancer is the leading cause of cancer death in women in developed countries. Staging is crucial in determining the treatment modality. Different treatment modalities for lung cancer include surgery, radiation therapy, and chemotherapy. PET/CT is the most sensitive and accurate modality for detecting hilar, mediastinal, and metastatic disease except in the brain. PET/CT scan often upstages the disease. PET/CT has also been shown to have false positive results. We present the case of a 72-year-old female who had a false positive finding on PET/CT, which would have changed the management process and outcome of her disease.

## Introduction

Lung cancer is the leading cause of cancer women's death in developed regions of the world [1]. Lung cancer treatment has evolved throughout the years. Currently, the stage of the disease determines treatment [2]. Lung cancer staging involves the TNM classification, which takes into account tumor size, endobronchial location, local invasion, separate tumor nodule(s), lymph node involvement, and metastases [3]. Fluorodeoxyglucose (FDG)-positron emission tomography (PET) computed tomography (CT) is the single most sensitive and accurate modality for detecting hilar and mediastinal involvement as well as detecting metastatic disease except for in the brain [4]. PET/CT has changed management in non-small cell lung cancer in 30%-40% of cases [5] and often upstages the disease [6]. We present the case of a 72-year-old female with non-small cell lung cancer, and her PET/CT falsely upstaged her disease, and the biopsy established the correct stage and treatment plan. 

## Case presentation

**Figure 1:**
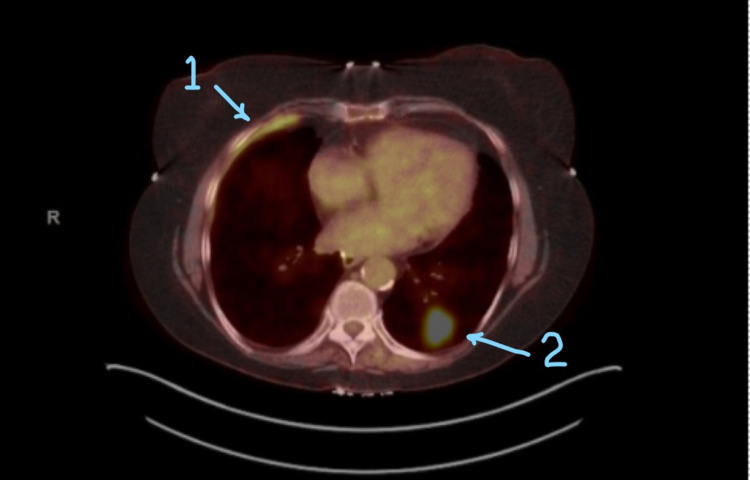
Hypermetabolic nodule (2) with spiculated margins in the left lower lobe and hypermetabolic pleural thickening (1) in the right lower lobe suspicious of malignancy 1. Hypermetabolic pleural thickening 2. Hypermetabolic nodule

This is a 72 y/o Caucasian female with a history of prior right pleural effusion of uncertain cause that occurred five years before her current presentation and 50 pack-year smoking history who presented with complaints of discomfort in her right chest. A CT scan of her chest was obtained, which revealed a spiculated pulmonary mass of the left lower lobe measuring 2.4x2.4 cm. The mass was biopsied and found to be invasive squamous cell carcinoma, moderately differentiated, and keratinizing (non-small cell lung cancer). The plan was made to proceed with staging workup and possibly left lower lobe lobectomy. A staging workup was initiated. PET/CT was also obtained, which showed a 2.2x2.5 cm hypermetabolic nodule with spiculated margins in the left lower lobe correlating to a previously identified nodule on CT. A hypermetabolic pleural thickening within the right lower lobe was also demonstrated on PET/CT, suspicious of malignancy (Figure 1).

We were concerned about a synchronous lesion in the contralateral lobe, which would have increased her lung cancer stage, changed management, and increased mortality risk. The decision was made to proceed with the right video-assisted thoracoscopic surgery with pleural biopsy to rule out metastatic disease or a second malignancy. The patient underwent the procedure without any adverse events. During the procedure correlating with the area in question on the PET CT scan, “dense adhesions and cottage cheese-like exudate” were seen throughout the major fissure and coursing posteriorly to the diaphragm with a nodule on the diaphragm. Biopsies were taken, and the exudate was removed. Pathology showed findings consistent with chronic inflammation without any infectious or malignant processes. After recovery, the patient was able to undergo definitive operative management with a left lower lobectomy. 

## Discussion

Staging workup of lung cancer includes first obtaining a CT chest with IV contrast and a PET-CT directed at potential metastasis sites. Patients need to undergo invasive staging when they have mediastinal lymph nodes greater than 1 cm, a tumor that is bulky and encircling/invading the mediastinal structures, or when patients have normal size mediastinal lymph nodes but are at elevated risk for nodal disease. The invasive biopsy includes tissue sampling of the mass and mediastinal lymph nodes [7]. 

Biopsies of the mass and mediastinal lymph node sampling revealed non-small cell lung cancer with no nodal disease, respectively. However, when we obtained a PET/CT demonstrated an area of concern in the contralateral lung. The PET/CT showed a hypermetabolic pleural thickening in the right lower lung with a maximum SUV of 5.84, which is suspicious of malignancy. A second lesion in the contralateral lung would change the stage of the disease and, therefore, the treatment plan. We felt that it was strange to have a site of metastasis in the opposite lung when the mediastinal lymph node sampling had been negative for malignancy. This prompted us to further investigate by sampling the area of concern on the right lower lobe. A biopsy of the right lung was negative for malignancy, so the patient underwent surgical resection via left lower lobectomy of the primary malignant lesion. 

The literature review showed PET/CT scan, when used as a diagnostic tool, to have a false positive rate of 6.5% [8] and a specificity of 70% when not combined with other imaging modalities and 75% when combined with other imaging modalities [9]. In our patient, the false positive result of the PET/CT would have completely changed management by precluding our patient from definitive operative intervention if the metastatic disease was present [10]. However, given no malignancy was seen on the biopsy of the right pleura, our patient was correctly staged at Stage IA3, and surgical resection of the cancer was able to be performed. Further investigation of the PET/CT positive right lower lobe pleura thickening through tissue biopsy changed this patient’s management. 

## Conclusions

We report a case of lung cancer in a 72-year-old female who was falsely upstaged by PET/CT scan. A biopsy of the hypermetabolic pleural thickening of the right lower lobe of her lung showed no malignancy. This allowed our patient to proceed with definitive operative management for her lung cancer. The false positive result on PET/CT would have changed the management of this patient. Our patient was able to undergo operative intervention after reviewing and making decisions based on inconsistent results during lung cancer staging. 
